# Decomposition of the anisotropic strain in 3D-structure GaN layers using Raman spectroscopy

**DOI:** 10.1038/s41598-024-53478-2

**Published:** 2024-02-09

**Authors:** Kazuma Takeuchi, Hiroyuki Ogura, Noriyuki Hasuike, Takeshi Kamikawa

**Affiliations:** 1 Corporate R&D Group, Keihanna Research Center, Kyocera Corporation, 3-5-3 Hikaridai, Seika-cho, Soraku-gun, Kyoto, Japan; 2 Faculty of Electrical Engineering and Electronics, Kyoto Institute of Technology, Matsugasaki, Sakyo-ku, Kyoto, 606-8585 Japan

**Keywords:** Condensed-matter physics, Materials for devices, Materials for optics, Lasers, LEDs and light sources, Optical materials and structures, Optical physics

## Abstract

Strain engineering for gallium nitride has been studied by many researchers to improve the performance of various devices (i.e., light-emitting diodes, laser diodes, power devices, high electron mobility transistors, and so on). Further miniaturization of gallium nitride devices will clearly continue in the future, and therefore an accurate understanding of the strain state in the devices is essential. However, a measurement technique for axially resolved evaluation of the strain in microareas has not yet been established. In this study, we revealed that the anisotropic strain state induced in c-plane growth gallium nitride is linked to the split state of Raman peaks, which were measured with $z(xx)\overline{z }$ and $z(yx)\overline{z }$ polarized configurations. The anisotropic strain state in c-plane gallium nitride was induced in the 3D-structure by epitaxial lateral overgrowth, which enabled successful performance of our work. This result allowed us to axially decompose the strain in c-plane gallium nitride through Raman spectroscopy and establish a measurement technique for axially resolving the strain. This measurement technique is feasible using a conventional Raman spectrometer. Furthermore, the method was indicated to be applicable to all wurtzite-type crystals, including gallium nitride, silicon carbide, and aluminum nitride. Our work provides a new perspective for understanding the complex strain state in microareas for wurtzite materials. Comprehending the strain state, which strongly affects device performance, will help promote the research and development of III-V semiconductor devices.

## Introduction

Wurtzite III-V nitride semiconductors have been broadly adopted as electronic and optical devices due to their high stability and direct and wide bandgap. Gallium nitride (GaN) has attracted particular attention because of its high electron mobility in addition to these properties^1–3^. Although GaN is a fascinating material, it has several problems. One of the problems is that bulk substrates are currently expensive. Therefore, heterogeneous substrates such as silicon (Si), silicon carbide (SiC), and sapphire are often used for fabrication of GaN devices. However, the mismatch of the lattice constant and the thermal coefficient between the GaN epitaxial film and the substrate induces strain and dislocations in the epitaxial film. These dislocations and defects cause performance degradation of the device; thus, many studies on strain relaxation in GaN epitaxial films have been performed^4–8^. However, effective application of strain in films has been reported to lead to improve device performance because of polarization and bandgap control^9–12^. Strain is an important factor for devices, and precise control of strain in epitaxial films is required for device development and manufacturing. In addition, the device size or shape is changed through the manufacturing process, which means that the strain in epitaxial films may also be changed: relaxation of the strain or a transition from isotropic to anisotropic strain could occur. The strain values along the *x*-, *y*-, and z-axes should be measured at some key points in the manufacturing process. Furthermore, understanding the strain state in microareas has become more important because the device size has decreased, such as in micro-light-emitting diodes (microLEDs) and micro electro mechanical systems (MEMSs)^8,12–14^. In addition, the 100 μm cavity GaN based edge emitting laser diodes (LDs) was fabricated by an automatic cleavage technique using anisotropic strain in microareas^15^, and an accurate understanding of the strain field is essential. In other words, a method for evaluating axially resolved strain in a microarea is essential for future research and development of III-V semiconductor devices.

Several techniques for strain measurement are well known: electron backscatter diffraction (EBSD), X-ray diffraction (XRD), transmission electron microscopy (TEM), and Raman spectroscopy. Among these techniques, those that can measure the strain in a microarea include EBSD, TEM and Raman spectroscopy. In particular, Raman spectroscopy is a very powerful tool for examining microdevices, the benefits of which include atmospheric, brief, nondestructive, and high-spatial-resolution measurements. However, there are few reports on evaluating the axially resolved strain of GaN by Raman spectroscopy. Multiaxial strain evaluation of m- and a-plane GaN was reported by Feng et al.^16,17^. In contrast, even though c-plane GaN is easier to grow than semipolar/nonpolar GaN and is widely used for devices, there are no reports on its multiaxial strain evaluation. The reason why multiaxial strain evaluation of c-plane GaN by Raman spectroscopy is not mature is speculated to be that c-plane GaN epitaxially grown on c-sapphire or Si(111) substrates has basically in-plane isotropic strain due to its hexagonal symmetry. Thus, axial decomposition of the in-plane strain has not been necessary. As mentioned above, c-plane GaN structures with microscale and complex shapes such as MEMSs and nanowires might have in-plane strain with broken isotropy. Raman measurements of c-plane GaN with in-plane anisotropic strain were reported by Darakchieva et al.^18^. Regarding Raman measurement of anisotropically strained GaN, Darakchieva et al. demonstrated that E_2_(high) Raman peak splitting was observed under two different polarization configurations. In addition, a larger peak splitting was indicated to correspond to a greater anisotropy ratio of the in-plane strain. However, the anisotropic strain was not axially decomposed in the paper.

In this study, we established a measurement technique for axially decomposing the anisotropic strain in c-plane GaN by Raman spectroscopy. To establish the technique, c-plane GaN samples with simple uniaxial strain were prepared. The strain values obtained by Raman spectroscopy were compared with the results calculated from XRD. Our research focused on characterizing the complex anisotropic strain state in c-plane GaN which strongly affects the device performance, and it undoubtedly elucidates the further promotion of research, development, and fabrication of nitride semiconductor devices.

## Results

### Polarized Raman spectra of isotropic/anisotropic strain GaN

**Figure 1:**
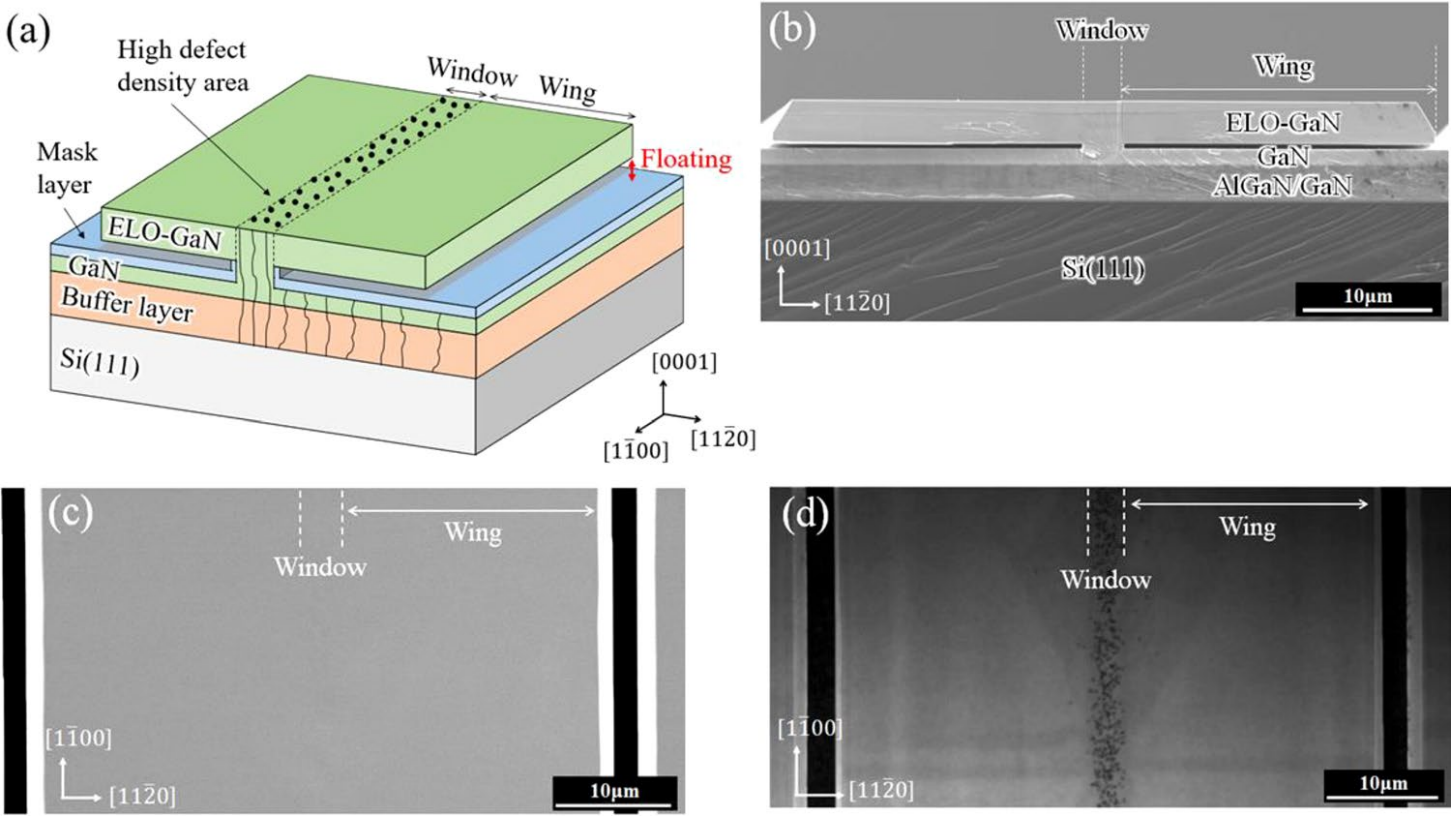
(**a**) Schematic diagram of the ELO-GaN layer on a Si(111) substrate, SEM images of an ELO-GaN layer on a Si(111) substrate for the (**b**) cross section and (**c**) top surface, and (**d**) CL image of the top surface.

Anisotropically strained c-plane GaN samples were grown on Si and sapphire substrates using the epitaxial lateral overgrowth (ELO) technique^19^. A schematic diagram of ELO-GaN is shown in Fig. 1a, and scanning electron microscopy (SEM) images of ELO-GaN are shown in Fig. 1b and c. To induce anisotropic strain in c-plane GaN as simply as possible, our ELO-GaN has two features that are different from those of the conventional ELO layer. First, the wing region in ELO-GaN is floated above a mask layer, which is a no-growth area. Second, the edges of the wing regions grown laterally from adjacent stripes do not coalesce. Both features prevent unintended stress in the wing region caused by contact with the mask or the adjacent wing. The stress induced in the wing region was generated from the window region, which was epitaxially grown on the heterogeneous underlayer. The window region had a length of approximately 10 mm and a width of 3.5 μm, and the length was extremely large compared to the width. In this case, we expected the stress along the width, $\left[11\overline{2 }0\right]$, direction to be smaller than that along the length, $\left[1\overline{1 }00\right]$, direction because the width of the stripes was divided by a shorter distance. Thus, the strain state in ELO-GaN may be simple, and we anticipated that much of the strain induced in the wing region would be along the stripes: uniaxial strain along the $\left[1\overline{1 }00\right]$ direction. One of the measurement techniques for threading dislocations in GaN crystals is cathodoluminescence (CL)^20,21^. Threading dislocations are observed as dark spots in CL imaging. However, note that CL underestimated the dislocation density typically by one order of magnitude. As shown in Fig. 1d, the dark spots were concentrated in the window region, where the threading dislocation density was estimated to be approcimately 6.6 × 10^8^/cm^2^ by counting the number of dark spots. However, no dark spots were observed in the wing region. For this reason, we considered that there were few threading dislocations in the wing region. In other words, the wing region in ELO-GaN was a uniaxially strained single-crystal GaN substrate.

**Figure 2:**
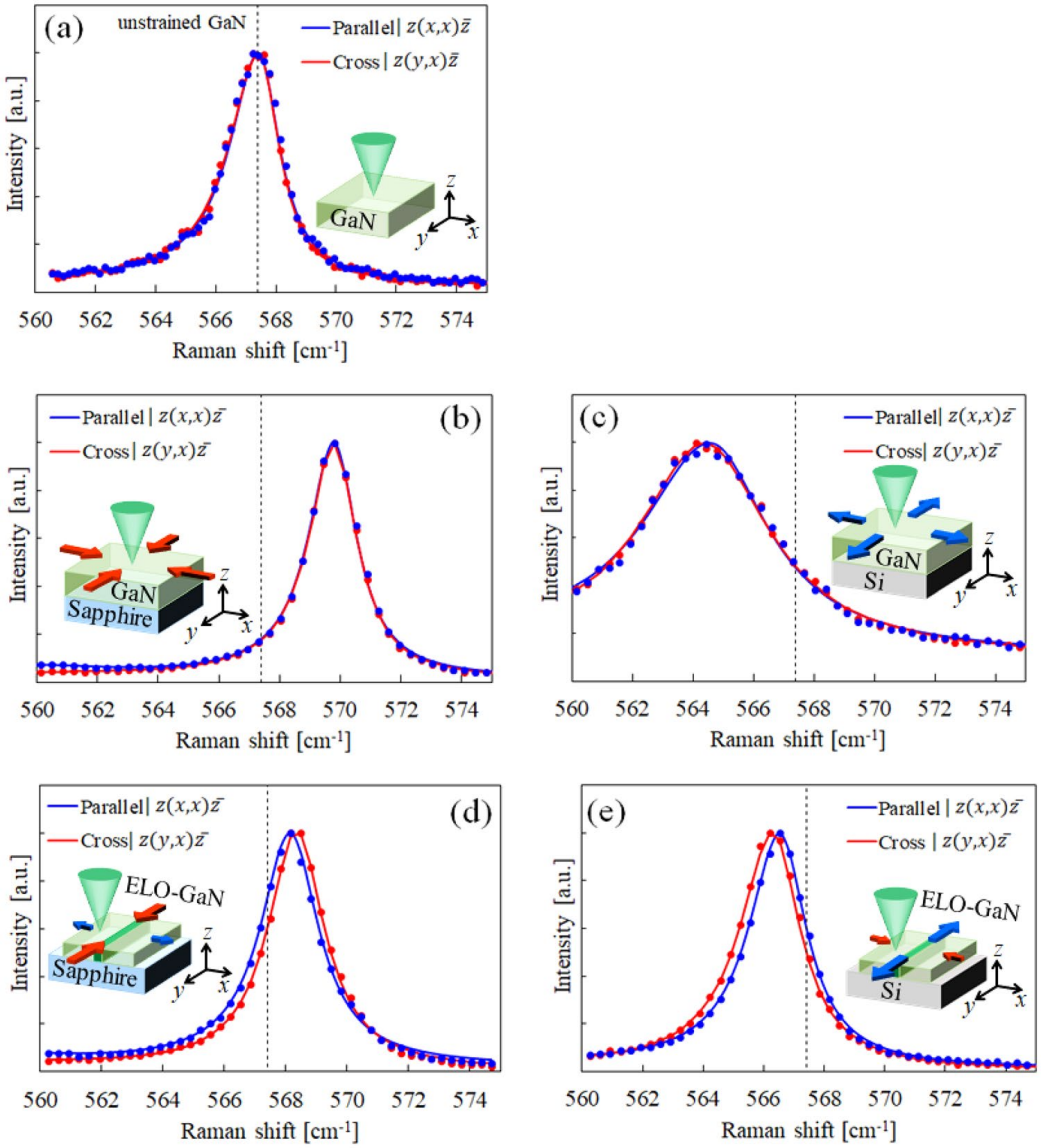
Polarized Raman spectra for the E2(high) phonon from the (**a**) free-standing GaN substrate, (**b**) GaN layer on the c-plane sapphire substrate, (**c**) GaN layer on the Si(111) substrate, (**d**) ELO-GaN layer on the c-plane sapphire substrate, and (**e**) ELO-GaN layer on the Si(111) substrate. The dashed line represents the 567.4 cm^-1^ Raman shift for unstrained GaN. The Raman spectra were obtained from flat-GaN layers and from the center of the wing region in the ELO-GaN layers.

According to Darakchieva et al., for c-plane GaN with in-plane anisotropic strain, the E_2_(high) mode Raman peaks obtained under $z(xx)\overline{z }$ (parallel) and $z(yx)\overline{z }$ (cross) polarization configurations are separated^18^. $z(xx)\overline{z }$ and $z(yx)\overline{z }$ are in Porto notation, and *x*, *y*, and *z* represent the $\left[11\overline{2 }0\right]$, $\left[1\overline{1 }00\right]$, and [0001] direction, respectively. Although, there was no mention of a free-standing GaN substrate and isotropically strained GaN layers in reference 17, the two Raman spectra obtained under their polarization configurations are anticipated to overlap completely. To study in detail the behavior of the E_2_(high) phonon in isotropically and anisotropically strained GaN crystals, five types of samples with different strain states were measured by polarized Raman spectroscopy: a free-standing c-plane GaN substrate, a GaN layer on a Si(111) substrate, a GaN layer on a c-plane sapphire substrate, an ELO-GaN layer on a Si(111) substrate, and an ELO-GaN layer on a c-plane sapphire substrate. The strains induced in the GaN layers are caused by thermal expansion coefficient mismatch with the Si(111) or c-plane sapphire substrate. The coefficient of GaN is larger than that of Si and smaller than that of sapphire ^22^. Thus, tensile strain is induced in the GaN layer on the Si(111) substrate, and compressive strain is induced in the GaN layer on the c-plane sapphire substrate. The ELO-GaN layers on the Si(111) and c-plane sapphire substrates exhibit essentially the same behavior. However, they are considered to have uniaxial strain, as described in the previous section. As shown in Fig. 2, for each E_2_(high) peak obtained under the parallel and cross configurations, the peaks from the free-standing c-plane GaN substrate and the GaN layers coincided, whereas those from the ELO-GaN layers were separated. This result suggests that the flat-GaN layers had isotropic strain, while the ELO-GaN layers had anisotropic strain. This peak splitting indicates that the double degeneracy of the E_2_(high) phonon was lifted, which is presumably due to optical anisotropy caused by in-plane anisotropic strain. The Raman peak is determined by the maximum of the imaginary part Im(− 1/*ε*) in the energy loss function, where *ε* is the complex dielectric function^24–26^. The complex dielectric function is closely related to the optical constants. Therefore, the variation in the Raman peak position implies variation of the optical constant. In fact, Darakchieva et al.^18^ measured the complex dielectric functions for the $\left[11\overline{2 }0\right]$ and $\left[1\overline{1 }00\right]$ directions of c-plane GaN with in-plane anisotropic strain by a spectroscopic ellipsometer and demonstrated that the respective peak positions were separated. In contrast, for c-plane GaN with in-plane isotropic strain, these peak positions are expected to coincide.

The Raman peaks measured from the anisotropically strained GaN layers in Fig. 1 were at 566.6 cm^−1^ (parallel configuration) and 566.3 cm^−1^ (cross configuration) for the ELO-GaN layer on the Si(111) substrate, and at 568.1 cm^−1^ (parallel configuration) and 568.4 cm^−1^ (cross configuration) for the ELO-GaN layer on the c-plane sapphire substrate. The spectral resolution of the Raman spectrometer is 0.3 cm^−1^ with a measurement error of ± 0.01 cm^−1^. Therefore, we considered that the Raman peak splitting obtained for each polarization configuration was significant. The obtained E_2_(high) spectrum contains a mixture of Raman signals from ELO-GaN and flat GaN underlayer. However, the depth of focus was less than 1.0 μm (See “Materials and Methods” for details.), which was sufficiently shallow compared to the thickness of the ELO-GaN layer. Therefore, the Raman spectrum was dominated by the ELO-GaN signal, and the Raman signal from the flat GaN sublayer was buried and hidden in the ELO-GaN signal. However, in order to rigorously perform the peak analysis of the ELO-GaN layer, even the very slightly observed underlying GaN signal was incorporated into the fitting analysis. Spectrum of only the flat GaN underlayer was obtained and its information (peak position, FWHM, and intensity) was incorporated to perform the spectral fitting (See “Supplementary information” for details.). In addition, the strain values in the $\left[11\overline{2 }0\right]$ and $\left[1\overline{1 }00\right]$ directions obtained by XRD were -0.05% and + 0.14% for the ELO-GaN layer on the Si(111) substrate, and + 0.02% and -0.13% for the ELO-GaN layer on the c-plane sapphire substrate, respectively. These results are summarized in Table 1. As a comparison, the c-plane GaN layer on an *a*-sapphire substrate was taken from reference 17. The Raman peaks obtained under the parallel and cross configurations are denoted as ${\omega }_{parallel}$ and ${\omega }_{cross}$, respectively, whose difference $\left|{\omega }_{parallel}-{\omega }_{cross}\right|$ represents the peak splitting magnitude. The magnitude of $\left|{\omega }_{parallel}-{\omega }_{cross}\right|$ indicates the anisotropy degree for the in-plane strain. Furthermore, the strain values in the $x:\left[11\overline{2 }0\right]$ and $y:\left[1\overline{1 }00\right]$ directions for anisotropically strained GaN were measured by XRD, and the strain state in the hexagonal crystal is shown in a schematic diagram that contains dashed lines (unstrained state) and colored lines (anisotropically strained state). The method for calculating strain values based on XRD is described in detail in the “Materials and methods“. Notably, the polarized Raman results were obtained from the wing region in ELO-GaN due to the smaller spot size, approximately ϕ1.0 μm, whereas the XRD results were averages of several ELO stripes measured together due to the larger spot size, approximately 5.0 × 5.0 mm. As shown in Fig. 1, ELO-GaN has wing and window regions, which may have different strain states because ELO-GaN is connected to the underlying substrate only in the window region. However, since the volume of the wing region is more than 10 times larger than that of the window region, the strain results obtained by XRD can be estimated to be mostly from the wing region. For the results shown in Table 1, the ${\omega }_{parallel}$ peak appeared at a higher wavenumber than the ${\omega }_{cross}$ peak for the ELO-GaN layer on the Si(111) substrate. In contrast, the opposite was true for the ELO-GaN layer on the sapphire substrate. This is expressed in the table by ${\omega }_{parallel}-{\omega }_{cross}$. The compressive or tensile in-plane strains (${\varepsilon }_{xx}$ and ${\varepsilon }_{yy}$) were similarly reversed for ELO-GaN on Si and sapphire substrates. The thermal expansion coefficients of GaN, Si, and sapphire have the following magnitude relationship, sapphire > GaN > Si^22^. Therefore, the GaN layer grown on the Si substrate is under induced tensile strain, while that on the sapphire substrate is under induced compressive strain. The XRD strain results were reasonable because the strains induced in GaN from the Si and sapphire substrates were tensile and compressive, respectively. A compressive strain was also induced in the flat c-plane GaN on a-sapphire for the same reason. Contrary to this, the sign of ${\omega }_{parallel}-{\omega }_{cross}$ for the flat c-plane GaN on *a*-sapphire was not consistent with that of ELO-GaN on sapphire but rather consistent with that of ELO-GaN on Si. This result indicated that the relationship between the positions of the ${\omega }_{parallel}$ and ${\omega }_{cross}$ peaks is independent of whether the strain induced in the GaN layer is compressive or tensile. It is correlated with the magnitude relationship between ${\varepsilon }_{xx}$ and ${\varepsilon }_{yy}$, which is represented in the schematic diagram of the hexagonal strain state. ${\varepsilon }_{yy}>{\varepsilon }_{xx}$ when ${\omega }_{parallel}>{\omega }_{cross}$, and the converse is also true. In other words, this result means that the shape of the hexagonal crystal can be easily determined by simply checking the positions of the ${\omega }_{parallel}$ and ${\omega }_{cross}$ peaks. This discovery led us to establish an evaluation method for the anisotropic strain in c-plane GaN using Raman spectroscopy.

**Table 1:** Polarized Raman and XRD strain measurements of ELO-GaN on Si, ELO-GaN on sapphire, and GaN on a-sapphire^18^. The measurement error of the Raman measurement was ± 0.01 cm^−1^.

	ELO-GaN on Si	ELO-Gan on *c*-Sapphire	*c*-GaN on *a*-Sapphire ^18^
By Raman spectroscopy			
*ω*_parallel_ [cm^−1^]	566.6	568.1	570.4
*ω*_cross_ [cm^−1^]	566.3	568.4	569.9
*ω*_parallel_ − *ω*_cross_ [cm^−1^]	+ 0.3	− 0.3	+ 0.5
By XRD			
*ε*_*xx*_ (*a*-axis strain)	− 0.05%	+ 0.02%	− 0.23%
*ε*_*yy*_ (*m*-axis strain)	+ 0.14%	− 0.13%	− 0.16%
Strain schematic (Dash line is unstrained GaN)			

### Derivation of the Raman frequency-to-strain conversion equation

In general, the conversion equation between Raman frequency *ω*_E2_ and strain for the E_2_ mode is given by

1 $${\omega }_{E2}-{\omega }_{0}={a}_{E2}\left({\varepsilon }_{xx}+{\varepsilon }_{yy}\right)+{b}_{E2}{\varepsilon }_{zz}\pm {c}_{E2}{\left[{\left({\varepsilon }_{xx}-{\varepsilon }_{yy}\right)}^{2}+4{\varepsilon }_{xy}^{2}\right]}^\frac{1}{2},$$

where *ω*_0_ is the Raman shift of unstrained GaN (567.4 cm^−1^), *a*_E2_, *b*_E2_, and *c*_E2_ are the phonon deformation potentials, *ε*_*xx*_, *ε*_*yy*_, and *ε*_*zz*_ are the strains along each axial direction, and *ε*_*xy*_ is the shear strain^23,27^. Since these directions are parallel to the crystal principal axes, the shear strain component is zero^18^. Equation (1) can be rewritten as follows:

2 $${\omega }_{E2}-{\omega }_{0}={a}_{E2}\left({\varepsilon }_{xx}+{\varepsilon }_{yy}\right)+{b}_{E2}{\varepsilon }_{zz}\pm {c}_{E2}\left|{\varepsilon }_{xx}-{\varepsilon }_{yy}\right|$$

In Eq. (2), the plus-minus sign in the third term on the right side represents the Raman peak splitting of the E_2_ phonon mode. Since the absolute value of the difference between *ε*_*xx*_ and *ε*_*yy*_ is included, whether *ε*_*xx*_ or *ε*_*yy*_ is larger must be known in advance. For this reason, Eq. (2) lacks versatility, which is probably why the evaluation of the anisotropic strain in c-plane GaN has not matured until now. However, the results in Table 1 link the position relationship between the ${\omega }_{parallel}$ and ${\omega }_{cross}$ peaks with the magnitude relationship between *ε*_*xx*_ and *ε*_*yy*_. Therefore, applying the relationship to the above, the following equations were obtained:

3 $${\omega }_{E{2}_{{\text{cross}}}}-{\omega }_{0}={a}_{E2}\left({\varepsilon }_{xx}+{\varepsilon }_{yy}\right)+{b}_{E2}{\varepsilon }_{zz}+{c}_{E2}\left({\varepsilon }_{xx}-{\varepsilon }_{yy}\right),$$

4 $${\omega }_{E2\_{\text{parallel}}}-{\omega }_{0}={a}_{E2}\left({\varepsilon }_{xx}+{\varepsilon }_{yy}\right)+{b}_{E2}{\varepsilon }_{zz}-{c}_{E2}\left({\varepsilon }_{xx}-{\varepsilon }_{yy}\right).$$

The stress along the growth direction of the c-plane films vanishes because the dangling bonds on the surface are free to expand or contract. Thus, the strain along the growth direction,* ε*_*zz*_, can be expressed using the two in-plane strain components as

5 $${\varepsilon }_{{\text{zz}}}=-\frac{{C}_{13}}{{C}_{33}}\left({\varepsilon }_{xx}+{\varepsilon }_{yy}\right),$$

where *C*_13_ and *C*_33_ are elastic stiffness constants. By substituting Eq. (5) into Eqs. (3) and (4), the Raman shifts are linked with the in-plane strain components as

6 $${\omega }_{E2\_{\text{cross}}}-{\omega }_{0}=\left({a}_{E2}-{b}_{E2}\frac{{C}_{13}}{{C}_{33}}\right)\left({\varepsilon }_{xx}+{\varepsilon }_{yy}\right)+{c}_{E2}\left({\varepsilon }_{xx}-{\varepsilon }_{yy}\right),$$

7 $${\omega }_{E2\_{\text{parallel}}}-{\omega }_{0}=\left({a}_{E2}-{b}_{E2}\frac{{C}_{13}}{{C}_{33}}\right)\left({\varepsilon }_{xx}+{\varepsilon }_{yy}\right)-{c}_{E2}\left({\varepsilon }_{xx}-{\varepsilon }_{yy}\right).$$

By solving Eqs. (6) and (7) as simultaneous equations, the in-plane strain in c-plane GaN can be axially decomposed from the measured Raman shifts. Equation (5) gives *ε*_*zz*_ from the calculated *ε*_*xx*_ and *ε*_*yy*_. There is no constraint (i.e., the magnitude relationship between *ε*_*xx*_ and *ε*_*yy*_ must be known in advance) on the above equations. Therefore, these equations are more versatile in evaluating the strain along each axis in c-plane GaN. For wurtzite GaN, the phonon deformation potentials *a*_E2_, *b*_E2_, and *c*_E2_ are − 850 ± 25 cm^−1^, − 920 ± 60 cm^−1^, and 65 ± 20 cm^−1^, respectively^28,29^. The elastic stiffness constants *C*_13_ and *C*_33_ are 93.4 GPa and 391.0 GPa, respectively^30^. The conversion equations between the Raman frequency and strain in c-plane GaN were obtained by substituting the respective constants into Eqs. (6) and (7) as follows:

8 $${\omega }_{E2\_{\text{cross}}}-567.4=-565.2{\varepsilon }_{xx}-695.2{\varepsilon }_{yy},$$

9 $${\omega }_{E2\_{\text{parallel}}}-567.4=-695.2{\varepsilon }_{xx}-565.2{\varepsilon }_{yy}.$$

**Figure 3:**
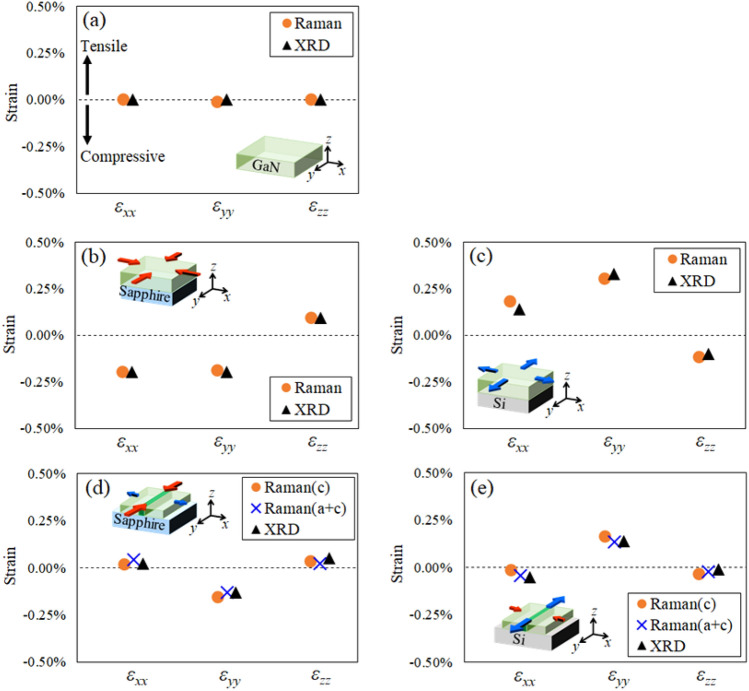
Strain results obtained by polarized Raman spectroscopy and XRD for the (**a**) free-standing GaN substrate, (**b**) GaN layer on the sapphire substrate, (**c**) GaN layer on the Si substrate, (**d**) ELO-GaN on the sapphire substrate, and (**e**) ELO-GaN on the Si substrate. The dashed line represents the unstrained state. Raman(c) and Raman(a + c) were calculated assuming stress release along the c-axis and along the a- and c-axes, respectively. The Raman spectra were obtained from bulk GaN, flat-GaN layers and the center of the wing region in the ELO-GaN layers. Errors in *ε*_*xx*_ and *ε*_*yy*_ were estimated to be approximately ± 0.10%, and those in *ε*_*zz*_ were estimated to be approximately ± 0.20%.

Using the derived conversion equation between the Raman frequency and strain, the strains in each axial direction were obtained from the Raman spectra shown in Fig. 2. Propagation errors in the measured values and substituted constants were considered in the calculation of the strain values. Errors in the in-plane strain values (*ε*_*xx*_ and *ε*_*yy*_) were estimated to be approximately ± 0.10%, and those in the out-of-plane strain values (*ε*_*zz*_) were estimated to be approximately ± 0.20%. The errors in the substituted constants accounted for the majority of these distortion errors. Determining more accurate constants will be a future work. They were then compared with the strain results from XRD, as shown Fig. 3. The polarized Raman and XRD strain results corresponded well for every GaN film in which different strain states were induced. The GaN layer on the c-plane sapphire substrate had an isotropic compressive strain. The GaN layer on the Si(111) substrate had an in-plane tensile strain state; however, the values of *ε*_*xx*_ and *ε*_*yy*_ differed, so it was not a strictly isotropic strain state. Anisotropic strain was perhaps induced in the c-plane GaN layer on the Si(111) substrate due to the dispersion of the GaN layer thickness or the bias of the substrate bowing. For the ELO-GaN layers on the Si(111) and c-plane sapphire substrates, the polarized Raman and XRD results also corresponded well. However, the validity of the cited phonon deformation potentials should be noted. The *a*_E2_ and *b*_E2_ obtained from GaN layers, which are assumed to be isotropically strained, are not strictly suitable for ELO-GaN layers with an anisotropic strain state. Therefore, we should derive *a*_E2_ and *b*_E2_ using anisotropically strained GaN, which is a subject for future work. Since the strain values of the ELO-GaN layer measured in this study were small, the difference from the XRD strain results should be small. The formation of the ELO-GaN layer on Si(111) and c-plane sapphire substrates induced contrary strains in each axial direction due to the mismatch of the thermal expansion coefficient with each substrate (e.g., the *ε*_*yy*_ of ELO-GaN on the Si(111) substrate is tensile strain, but that of ELO-GaN on the c-plane sapphire substrate is compressive strain). Interestingly, the *ε*_*yy*_ in the direction along the stripe corresponded to a relatively large tensile or compressive strain, whereas the *ε*_*xx*_ in the direction perpendicular to the stripe corresponded to the same amount of strain as the strain *ε*_*zz*_ in the out-of-plane direction. This result was considered to be due to the vanishing of the stress in the a-plane growth direction along the x-axis in the wing region floating above the mask layer. The vanishing of the stress was caused by the same reason as the vanishing of the stress along the c-axis in deriving Eq. (5), that is, the free expansion or contraction of the dangling bonds on the stripe side faces. In this case, not only the stresses in the c-plane but also those in the a-plane are released. For the released stress in the a-plane, the strain along the growth direction,* ε*_*zz*_, can be expressed as follows:

10 $${\varepsilon }_{{\text{zz}}}=-\frac{{C}_{11}}{{C}_{13}}{\varepsilon }_{xx}-\frac{{C}_{12}}{{C}_{13}}{\varepsilon }_{yy}.$$

From Eqs. (5) and (10), *ε*_*xx*_ can be derived as a linear function of *ε*_*yy*_ as follows:

11 $${\varepsilon }_{xx}=-\frac{{C}_{13}^{2}-{C}_{12}{C}_{33}}{{C}_{13}^{2}-{C}_{11}{C}_{33}}{\varepsilon }_{yy}.$$

Substituting Eq. (11) into Eqs. (6) and (7), the conversion equation between the Raman frequency and strain is established based on the Raman shift and *ε*_*yy*_ as follows:

12 $${\omega }_{E2\_{\text{parallel}}}-{\omega }_{E{2}_{cross}}=2{c}_{E2}\left(1+\frac{{{C}_{13}}^{2}-{C}_{12}{C}_{33}}{{{C}_{13}}^{2}-{C}_{11}{C}_{33}}\right){\varepsilon }_{yy},$$

where the elastic stiffness constant *C*_12_ is 132.1 GPa ^29^. The strain results obtained from Eq. (12) for ELO-GaN on Si(111) and c-plane sapphire substrates were added to Fig. 3d and e. For the sake of distinction, the strain values obtained from Eq. (8) and (9) were denoted “Raman(c)” and those from Eq. (12) were denoted “Raman(a + c)” in the figures. The strain value obtained from Eq. (12) was closer to the strain value from XRD. Therefore, Eq. (12) was considered to be suitable for the special geometry of ELO-GaN. The residual strain in ELO-GaN was induced by the lattice mismatch with the underlayer and the thermal expansion coefficient mismatch with the substrate, for the same reasons as in planar GaN layer. However, due to the stripe shape, ELO-GaN had a peculiar anisotropic distortion. The strain along the ELO stripe (m-axis strain) was induced by the lattice mismatch with the underlayer and the mismatch of thermal expansion coefficient with the substrate. The m-axis residual strain became relatively large because the window region was connected to the underlayer by a long distance. Since the wing was epitaxially grown on the window, the m-axis residual strain in the wing region was inherited from the window region. On the other hand, the strain across the stripes (a-axis strain) was relaxed. The ELO stripes were approximately 50 µm wide, and adjacent stripes were not in direct contact with each other, suggesting that the dangling bonds on the topmost surface of the $\left(11\overline{2 }0\right)$ plane were released. Therefore, under ideal conditions, the stress in the a-axis direction was fully relaxed. Also, the stress along the c-axis direction was relaxed for the same reason. Thus, the origin of the a-axis strain was only due to elastic deformation caused by m-axis strain. Noted that Eq. (12) is not applicable in the case of stress in the a-plane direction (e.g., when the wing region contacts or adheres to the mask layer during lateral growth, and when adjacent wings are in direct contact in each other). In such cases, Eqs. (8) and (9) should be applied. Equations (8) and (9) are considered generally applicable for c-plane grown GaN films, regardless of the geometry of GaN layers, GaN nanowires, or GaN-MEMS bridges^13,14,31^. From the above, the strain results obtained by completely different measurement methods corresponded, so we are confident that a method for evaluating multiaxial strain in c-plane GaN using polarized Raman measurement has been established.

## Discussion

For comprehension of the in-plane anisotropic strain in c-plane GaN, a method to evaluate the strain in each axial direction by measuring the E_2_(high) peak splitting using polarized Raman spectroscopy was demonstrated. This peak splitting indicates that the double degeneracy of the E_2_(high) phonon mode is lifted. However, this physical phenomenon is not a feature of GaN only. The E_2_(high) phonon mode is one of the transverse vibration modes of the wurtzite crystal structure and is excited not only in GaN but also in materials such as AlN, InN, CdS, ZnO and 6H-SiC. E_2_(high) peak splitting due to anisotropic strain is expected to be observed in these materials as well, and in fact, E_2_(high) peak splitting was reported for CdS^23^. In other words, we consider Eqs. (8) and (9) to be very versatile because they can be used to decompose the strain induced in a variety of c-plane grown materials. In addition, we believe that they can be used to evaluate the strains in ternary mixed crystals such as AlGaN and InGaN. However, note that their phonon deformation potentials and elastic constants may vary with the aluminum and indium concentrations, and studies to determine these values are not mature at present. AlGaN, InGaN, and AlN, along with GaN, have been used as materials for optical devices such as laser diodes (LDs) and for switching devices such as high electron mobility transistors (HEMTs). 6H-SiC, in contrast, has been used as a material for power devices. Anisotropic strain induced in the AlGaN or InGaN active layer in LDs affects the luminescence properties because the degeneracy between heavy and light holes is lifted^32^. AlN and 6H-SiC are used as channel materials in transistors^33,34^. Strain induced in the channel changes the energy band structure and affects the carrier mobility^35^. Therefore, the phonon deformation potential and elastic constants of these materials must be determined. The determination of these constants is imagined to further increase the importance of our derived equations.

In conclusion, we observed peak splitting of the E_2_(high) phonon mode for our ELO-GaN layer by polarized Raman spectroscopy; the ELO-GaN layer was stressed only in the m-axis direction and was very simple uniaxially strained GaN. By comparing several anisotropically strained GaN layers, we revealed that the position correlation between the Raman peaks (${\omega }_{parallel}$ and ${\omega }_{cross}$) obtained under two different polarization configurations was related to the magnitude correlation between the in-plane strains (*ε*_*xx*_ and *ε*_*yy*_). Derivation of a the versatile equation linking the E_2_(high) Raman shifts and the strain values along each axial direction was achieved for the first time. The strain values along each axis were obtained using the derived equation and were compared with the XRD strain results. Both strain results corresponded well. The ability of the derived equation to accurately evaluate the anisotropic strain in c-plane GaN was verified. Furthermore, since the basis of this evaluation technique is the peak splitting of the E_2_(high) phonon mode spectra coming from the wurtzite crystal structure, this evaluation technique can be applied to every wurtzite material. We believe that this evaluation method will bring about breakthroughs for the research and development of nitride semiconductor devices as a whole.

## Materials and methods

### Polarized Raman spectroscopy

A conventional Raman spectrometer was used for Raman measurements. The spectral resolution of the Raman spectrometer is 0.3 cm^−1^ with a measurement error of ± 0.01 cm^−1^, which was determined by repeatedly measuring the phonon band of Si at 520 cm^−1^. The focal length and grating groove were 250 mm and 3000 lines/mm, respectively. A Nd:YAG laser with a wavelength *λ* of 532 nm was used as the excitation source, and the laser spot size was approximately 0.7 μm when a 150 × objective lens with a numerical aperture (NA) of 0.95 was used. Thus, the depth of focus *d* was estimated to be approximately 0.6 μm using the following equation:

13 $$d=\lambda /{(NA)}^{2}$$

The parallel configuration $z(xx)\overline{z }$ and the cross configuration $z(yx)\overline{z }$ were realized by inserting polarizers on the incident and/or scattered light side.

### High-resolution X-ray diffraction

The high-resolution XRD system was equipped with a 4-circle goniometer and used a characteristic X-ray wavelength $\lambda$ of 1.5406 nm from a CuKα source. The incident beam was passed through a hybrid monochromator, which consisted of a parabolic graded multilayer mirror (X-ray mirror) and two channel-cut Ge(220) crystals. The diffraction beam from the samples was analyzed with high angular resolution by three channel-cut Ge(220) crystals. GaN strains in the $x:\left[11\overline{2 }0\right]$, $y:\left[1\overline{1 }00\right]$, and $z:\left[0001\right]$ directions were measured by 2*θ*-*ω* scanning of $\left(11\overline{2 }4\right)$, $\left(2\overline{2 }04\right)$, and (0002) diffraction, respectively. Calibration based on the bottom substrate eliminated mechanical errors. It was then confirmed that the mechanical errors were eliminated by measuring several diffraction planes at once or in sequence (see “Supplementary information” for details). The lattice spacing in each axial direction was obtained, and the strain values were calculated from the difference from the lattice spacing of strain-free GaN (calculated from a = 3.18 Å and c = 5.17 Å). The lattice spacing ${d}_{hklm}$ in each axial direction was obtained by substituting the measured $2{\theta }_{hklm}$ angle and incident angle ${\omega }_{hklm}$ into the following equations:

14a $${d}_{11\overline{2}0 }=\frac{\lambda }{{\text{cos}}{\omega }_{11\overline{2}4 }-{\text{cos}}\left(2{\theta }_{11\overline{2}4 }-{\omega }_{11\overline{2}4 }\right)}$$

14b $${d}_{2\overline{2}00 }=\frac{\lambda }{{\text{cos}}{\omega }_{2\overline{2}04 }-{\text{cos}}\left(2{\theta }_{2\overline{2}04 }-{\omega }_{2\overline{2}04 }\right)}$$

14c $${d}_{0002}=\frac{\lambda }{{\text{sin}}{\omega }_{0002}+{\text{sin}}\left(2{\theta }_{0002}-{\omega }_{0002}\right)}$$

The tilt angle of the crystallographic orientation of the floating ELO-GaN wing region was confirmed by measuring the rocking curve in the (0002) plane. We confirmed that one of the measured samples was slightly tilted in the $\left[11\overline{2 }0\right]$ or the $\left[\overline{1 }\overline{1 }20\right]$ direction, and the strain values were estimated by correcting for the tilt when determining the lattice parameter and strain by XRD^36^. The spot size varied with the incident angle of the X-ray beam and was 5.3 × 5.0 mm, 5.1 × 5.0 mm, and 3.4 × 5.0 mm for $\left(11\overline{2 }4\right)$, $\left(2\overline{2 }04\right)$, and (0002) diffraction, respectively.

### Supplementary Information

Supplementary Information 1.

Supplementary Information 2.

Supplementary Information 3.

Supplementary Information 4.

Supplementary Information 5.

## Data Availability

All data generated or analyzed during this study are included in this published article and its supplementary information files.
