# Supplementary figures and images for: Decomposition of the anisotropic strain in 3D-structure GaN layers using Raman spectroscopy
Source: Sci Rep. 2024 Feb 9;14:3330. doi: 10.1038/s41598-024-53478-2 (PMC10858272; doi:10.1038/s41598-024-53478-2)

## Slide 1
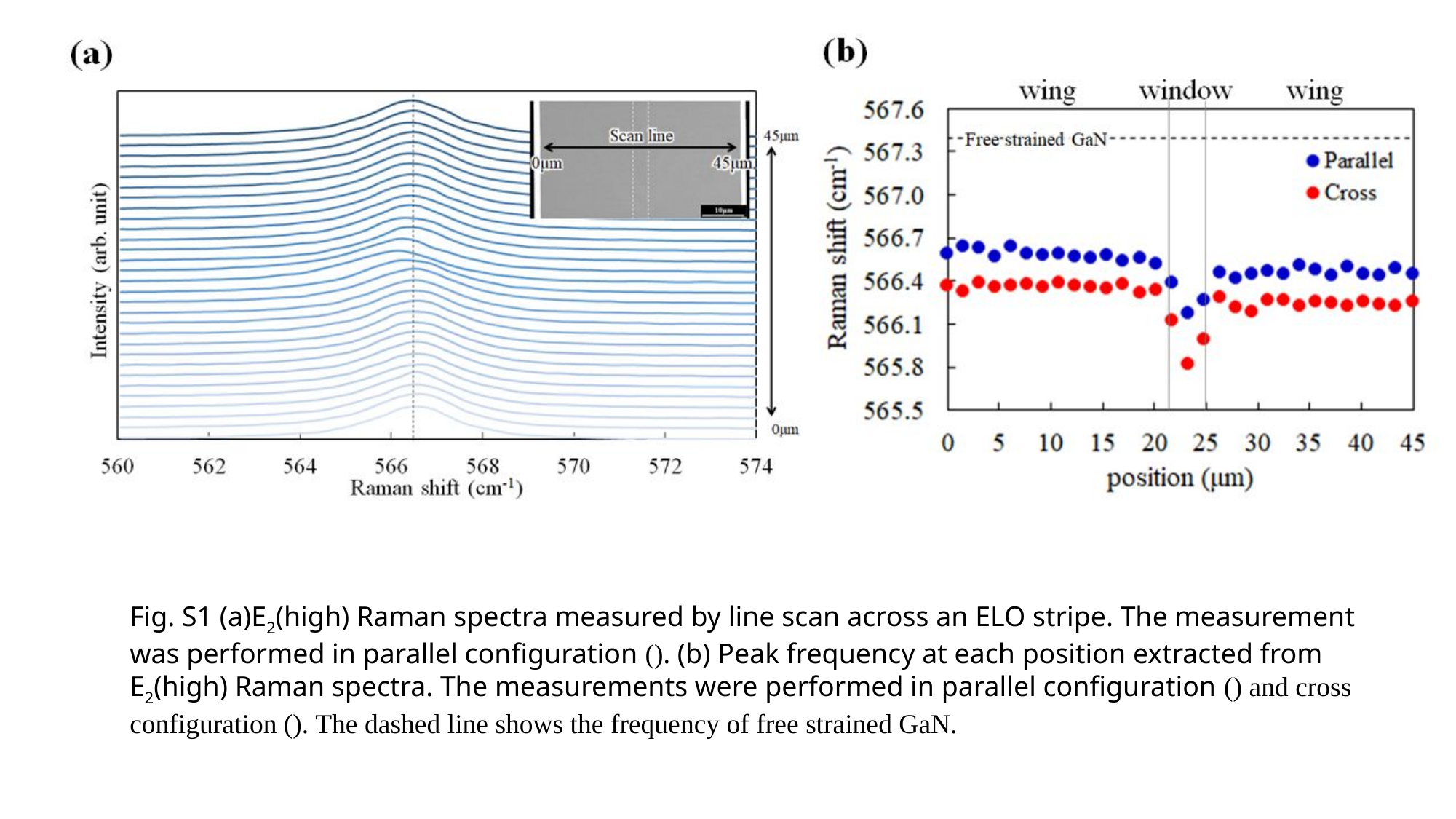

Supplement: Supplementary file 1 — Supplementary Information 1. [file 41598_2024_53478_MOESM1_ESM.pptx]

## Slide 1
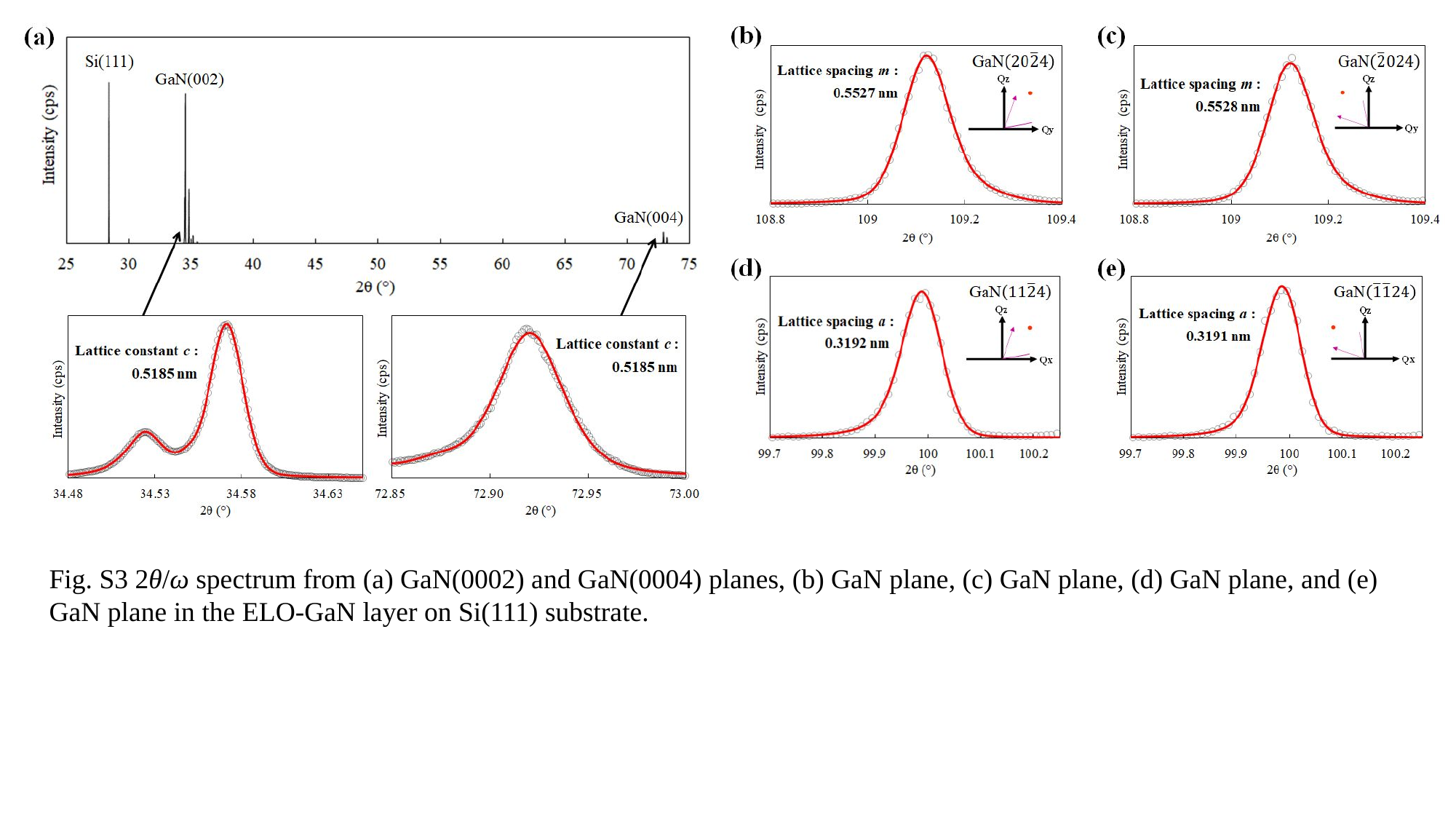

Supplement: Supplementary file 3 — Supplementary Information 3. [file 41598_2024_53478_MOESM3_ESM.pptx]
