# Supplementary material for: Decomposition of the anisotropic strain in 3D-structure GaN layers using Raman spectroscopy
Source: Sci Rep. 2024 Feb 9;14:3330. doi: 10.1038/s41598-024-53478-2 (PMC10858272; doi:10.1038/s41598-024-53478-2)
Supplement: Supplementary file 2 — Supplementary Information 2. [file 41598_2024_53478_MOESM2_ESM.pptx]

## Slide 1
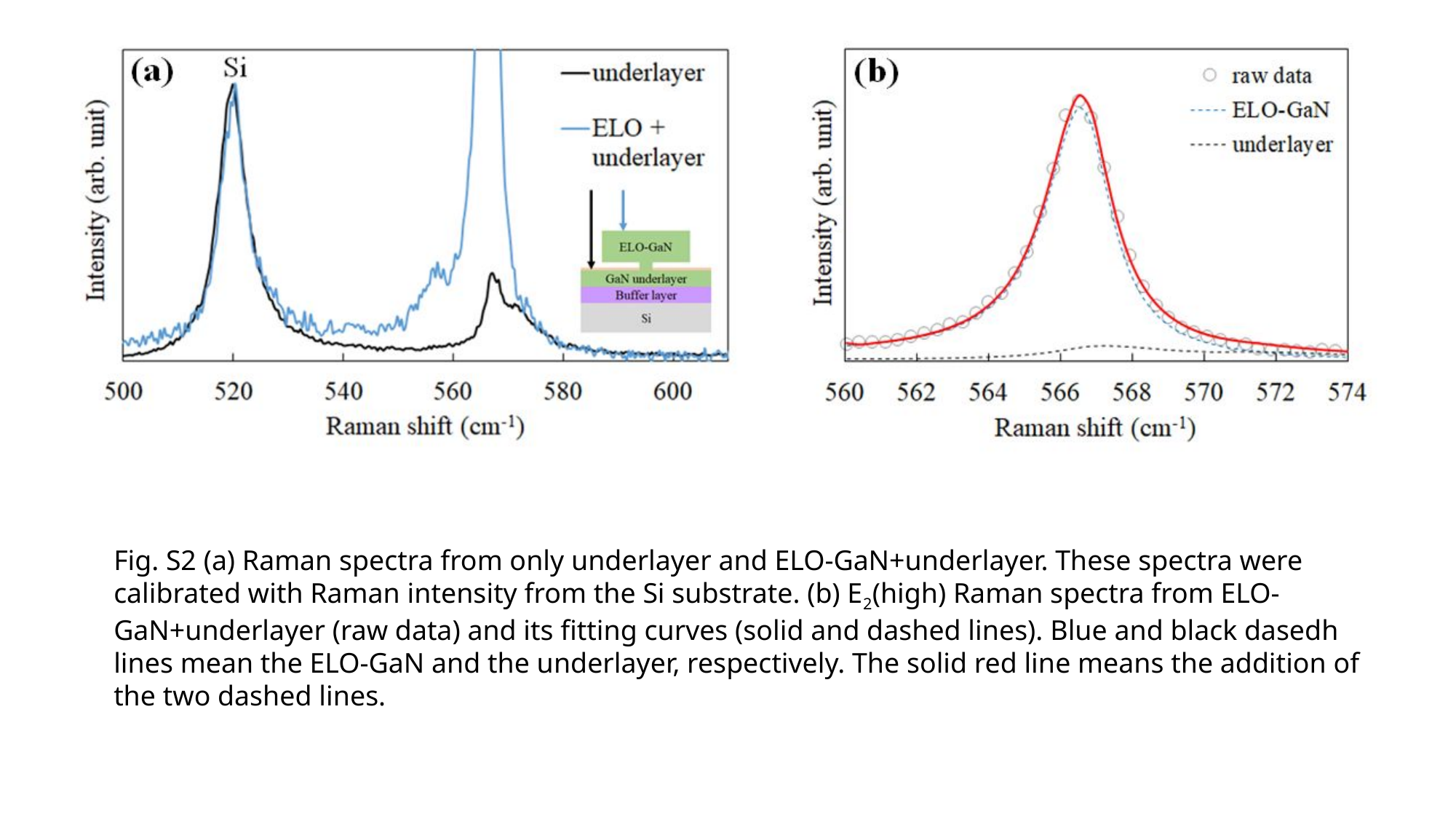

Fig. S2 (a) Raman spectra from only underlayer and ELO-GaN+underlayer. These spectra were calibrated with Raman intensity from the Si substrate. (b) E2(high) Raman spectra from ELO-GaN+underlayer (raw data) and its fitting curves (solid and dashed lines). Blue and black dasedh lines mean the ELO-GaN and the underlayer, respectively. The solid red line means the addition of the two dashed lines.
