# Supplementary material for: Decomposition of the anisotropic strain in 3D-structure GaN layers using Raman spectroscopy
Source: Sci Rep. 2024 Feb 9;14:3330. doi: 10.1038/s41598-024-53478-2 (PMC10858272; doi:10.1038/s41598-024-53478-2)
Supplement: Supplementary file 4 — Supplementary Information 4. [file 41598_2024_53478_MOESM4_ESM.pptx]

## Slide 1
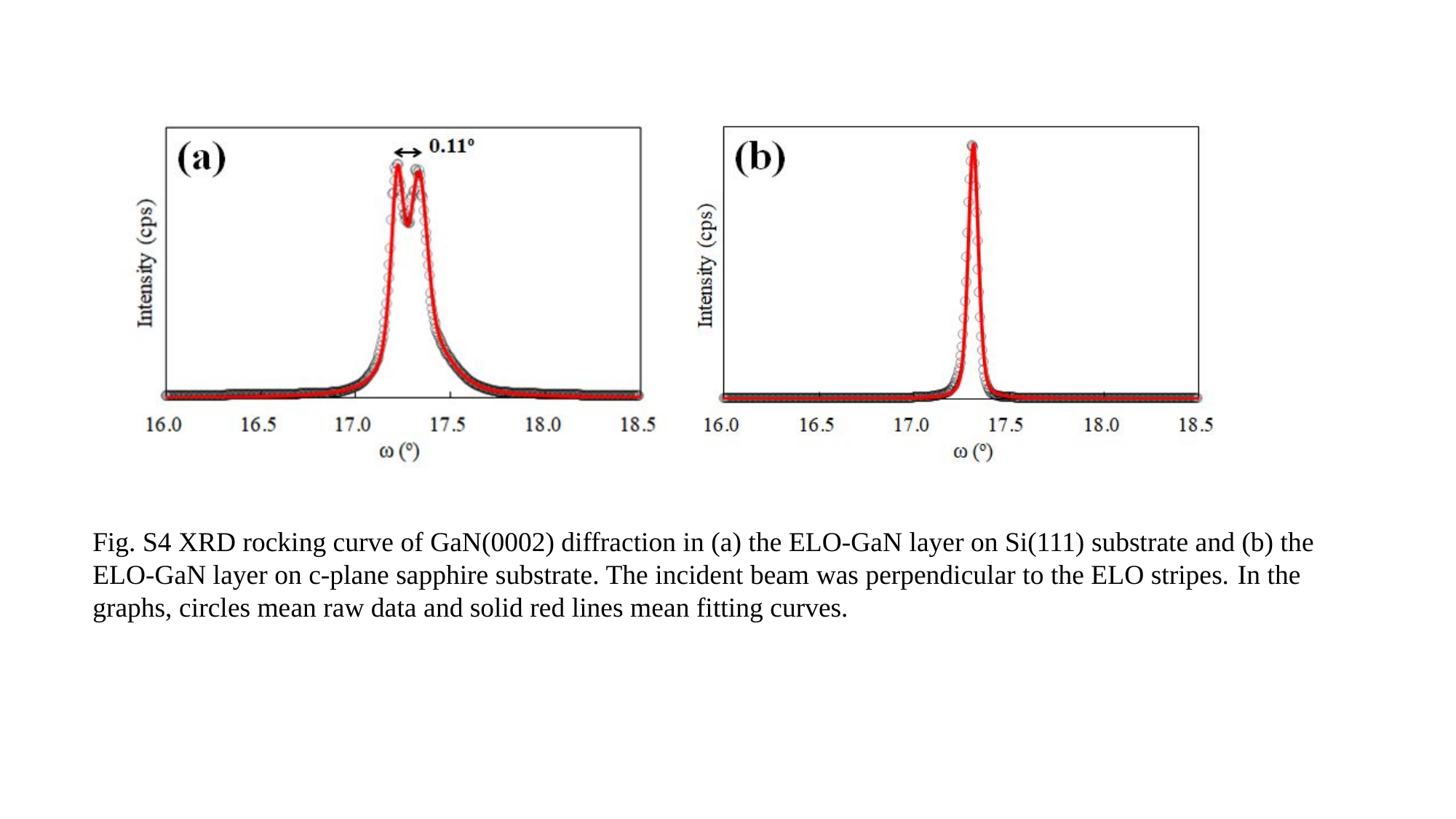

Fig. S4 XRD rocking curve of GaN(0002) diffraction in (a) the ELO-GaN layer on Si(111) substrate and (b) the ELO-GaN layer on c-plane sapphire substrate. The incident beam was perpendicular to the ELO stripes. In the graphs, circles mean raw data and solid red lines mean fitting curves.
