# Supplementary material for: Decomposition of the anisotropic strain in 3D-structure GaN layers using Raman spectroscopy
Source: Sci Rep. 2024 Feb 9;14:3330. doi: 10.1038/s41598-024-53478-2 (PMC10858272; doi:10.1038/s41598-024-53478-2)
Supplement: Supplementary file 5 — Supplementary Information 5. [file 41598_2024_53478_MOESM5_ESM.docx]

**Supplementary information**

**Decomposition of the Anisotropic Strain in 3D-Structure GaN Layers using Raman Spectroscopy**

Kazuma Takeuchi^1^, Hiroyuki Ogura^1^, Noriyuki Hasuike^2^, and Takeshi Kamikawa^1^

*^1^ Corporate R&D Group, Keihanna Research Center, Kyocera Corporation 3-5-3 Hikaridai Seika-cho Soraku-gun Kyoto, Japan*

*^2^ Department of Comprehensive Sciences, Kyoto Institute of Technology, Kyoto 606-8585, Japan*

*E-mail: kazuma.takeuchi.cy@kyocera.jp*

**Contents**

- Raman line scan across ELO-GaN stripe
- Curve fitting for Raman spectrum of GaN E_2_(high) vibration mode
- Calculation of strain value for ELO-GaN sample from XRD results

**Raman line scan across ELO-GaN stripe**

Raman line scan across an ELO-GaN stripe was performed on the ELO-GaN layers on Si substrate. In the line scan, the probe (spot diameter of approximately 1.0 um) was moved in 1.5 um increments to obtain Raman spectra of the E_2_(high) vibration mode from the ELO-GaN layer. The E_2_(high) Raman spectra obtained by parallel configuration ($z(xx)\bar{z}$) at each point are shown in Fig. S1(a) (cross configuration is not shown). The peak positions were extracted from these spectra and plotted in Fig. S1(b). For the ELO-GaN layers on Si substrate, the peak frequency of the E_2_(high) Raman spectra was constant at any point in the wing region. In addition, the peak splitting of the spectra obtained from the two configurations was also constant. These results suggest that uniform anisotropic strain was induced in the wing region. On the other hand, the peak splitting in the window region was larger than that in the wing region, which indicates that larger anisotropic strain was induced in the window region. This may be due to the fact that only the window region was grown epitaxially on the underlying substrate, which is a stressor.

**Curve fitting for Raman spectrum of GaN E_2_(high) vibration mode**

The spectra from the ELO-GaN samples obtained by Raman spectroscopy measurements include signals from the ELO-GaN layer and the underlying flat-GaN layer. Since the depth of focus of the excitation light was smaller than the thickness of the ELO-GaN layer, the signal from the underlayer was hidden by that of the ELO-GaN layer. In order to rigorously analyze the peak frequency of the Raman spectrum from the ELO-GaN layer, the E_2_(high) mode Raman spectrum from the underlayer only was obtained as shown in Fig. S2(a). Raman spectra from the ELO-GaN layer and the underlying layer were calibrated with Raman intensity from the Si substrate, respectively. Then, curve fitting of the Raman spectra from the ELO-GaN sample was performed taking into account the information of the underlayer as shown in Fig. S2(b). Curve fitting was performed by Lorentz function.

**Calculation of strain value for ELO-GaN sample from XRD results**

In the calculation of the lattice constants and strain values of the ELO-GaN layers by XRD measurements, a calibration based on the bottom substrate (Si or sapphire) of the ELO sample was performed to eliminate mechanical errors. For rigorous confirmation, diffractions from GaN(0002) and GaN(0004) planes were measured simultaneously. The result of 2θ/ω measurement in the ELO-GaN layer on the Si(111) substrate was shown in Figure S3(a). The c-axis lattice constants were calculated from the respective diffraction peaks using the miller index (*hkil*) and the lattice spacing d obtained by the Bragg equation (*λ*=2*d*sin*θ*). The lattice constants obtained from the diffraction peaks of the GaN(0002) and GaN(0004) planes were consistent. This result suggested that mechanical errors were eliminated. The diffraction measurements from the GaN$\left( 20\bar{2}4 \right)$ and GaN$\left( 11\bar{2}4 \right)$ planes were also performed after the same calibration as above. Diffraction measurements from GaN$\left( 20\bar{2}4 \right)$ and GaN$\left( 11\bar{2}4 \right)$ planes were performed in a setup with the sample tilt angle (psi or chi) of 0° for more precise analysis. In this setup, the laboratory system cannot measure the diffraction from GaN$\left( 10\bar{1}2 \right)$ and GaN$\left( 22\bar{4}8 \right)$ planes. Therefore, confirmation that mechanical errors were eliminated was performed using GaN$\left( \bar{2}024 \right)$ and GaN$\left( \bar{1}\bar{1}24 \right)$ planes. The diffraction measurements from GaN$\left( 20\bar{2}4 \right)$ and GaN$\left( \bar{2}024 \right)$ planes were performed in a row, and the same is true for GaN$\left( 11\bar{2}4 \right)$ and GaN$\left( \bar{1}\bar{1}24 \right)$ planes. The results were shown in Figure S3(b)-(d). The values of m-plane spacing along the ELO stripe or a-plane spacing across it, calculated from the respective peak positions, were displayed in the graph. Both values were in good match, clearly indicating that mechanical errors were eliminated from the results.

Since the wing region in the ELO-GaN layer was floating over the mask layer, there was a possibility of tilt in the crystallographic orientation. The tilt was observed by XRD measurements. The incident beam was perpendicular to the ELO stripes, and GaN(0002) rocking curves were obtained after calibration with the substrate. Since the ELO-GaN layer was thicker and more crystalline than the flat-GaN underlayer, the ELO-GaN layer was expected to obtain a larger diffraction signal. Therefore, if the crystallographic orientation in the wing region is tilted, some diffraction peaks from the ELO-GaN layer and the underlayer should be observed. On the other hand, if the crystallographic orientation in the wing region exactly corresponds to that of the underlayer, these diffraction peaks will be superimposed and a single peak will be observed. Figure S4(a) and (b) show GaN(0002) rocking curves from ELO-GaN layers on Si and sapphire substrates. The wing region in the ELO-GaN layer on c-plane sapphire substrate shows no crystallographic orientation tilt, while the wing region in the ELO-GaN layer on Si substrate is observed to have a crystallographic orientation tilt to approximately 0.06°. In calculating the a-axis strain (tilt direction), the reciprocal space coordinates Qx and Qz obtained from the measurement were corrected by rotating them by 0.06° (ref. 1).

**Reference**

[1] J. Kim, S. Lee, J. Oh, J. Ryu, Y. Park, S. –H. Park, and E. Yoon, Sci Rep 9, 8282 (2019). DOI: doi.org/10.1038/s41598-019-44519-2
